# Necrotising fasciitis: a fatal case of sepsis and a diagnostic challenge – case report and review of literature

**DOI:** 10.1186/s12245-018-0183-x

**Published:** 2018-04-06

**Authors:** Lekha Agarwal, Athar Yasin

**Affiliations:** Emergency Medicine Department, Peterborough City Hospital, North West Anglia NHS Foundation Trust, Peterborough, UK

**Keywords:** Necrotising fasciitis, Sepsis, Soft tissue infection, Rapid tissue necrosis

## Abstract

**Background:**

Necrotising fasciitis (NF) is a severe, devastating soft tissue infection characterised by rapidly progressing tissue necrosis. This rare condition has a high mortality rate and poses diagnostic and management challenges to the clinician. There is usually a history of trauma, which maybe trivial. Some of the premorbid conditions associated with NF are diabetes and or immunocompromised state. It requires prompt recognition and early treatment with intravenous antibiotics and extensive surgical debridement.

**Case presentation:**

We describe a 74-year-old lady who presented to our emergency department following 3 days’ history of watery diarrhoea and feeling generally unwell.

She had signs of severe sepsis and was started on broad-spectrum intravenous antibiotics and fluids for sepsis with unknown source. She was found to have an area of blackish discolouration on her thigh which was suspected as necrotising fasciitis (NF) and referred to the surgeons. She had no history of trauma or significant comorbidity. She underwent surgical exploration and debridement within few hours of arrival into the emergency department and subsequent further debridement with above-knee amputation of the affected limb.

She eventually died after about 48 h of hospital stay despite an early diagnosis and prompt surgical debridement and a multidisciplinary approach.

**Conclusions:**

Necrotising fasciitis has been previously reported in literature but we would like to highlight through this case the importance of looking for the source of sepsis by thorough clinical examination and the need to have a high threshold of suspicion for this rare condition and urgent involvement of a surgical team for debridement.

## Background

Necrotising fasciitis (NF) is a severe, devastating soft tissue infection characterised by rapidly progressing tissue necrosis. This rare condition has a high mortality rate and poses diagnostic and management challenges to the clinician. There is usually a history of trauma, which maybe trivial. Some of the premorbid conditions associated with NF are diabetes and or immunocompromised state. It requires prompt recognition and early treatment with intravenous antibiotics and extensive surgical debridement.

The infection may start along the fascial plane and cause symptoms including erythematous, painful and oedematous skin lesions, which often rapidly deteriorate to haemorrhagic blisters, anaesthesia, and gangrenous necrosis over several days. This process is usually accompanied by systemic toxic manifestations such as septic shock, reduced level of consciousness and multi-organ failure.

NF has been previously reported in literature but we would like to highlight through this case the importance of looking for the source of sepsis by thorough clinical examination and the need to have a high threshold of suspicion for this rare condition and urgent involvement of a surgical team for debridement.

## Case presentation

A 74-year-old lady presented to our emergency department following 3 days’ history of watery diarrhoea and feeling generally unwell. She had been ‘off legs’ for 3 days and was not improving hence called the GP for a home visit. She was referred to the hospital as a case of severe sepsis for medical admission and pre-alerted into Resus by paramedics. She denied any cough, cold, or urinary symptoms. She had a past medical history of hypertension and CKD and was on Ramipril and Atorvastatin. She was at reasonably normal baseline health and 3 days back started with watery diarrhoea. There was no history of recent travel and no vomiting or per rectal bleed. On examination, she looked unwell, still responding to verbal commands, hypotensive at 80/50 mmHg, and tachycardic at 110 beats per minute with a temperature of 38.5 °C. She was hypoxic on air, with saturations of 90% on high flow oxygen. She had evidence of peripheral cyanosis and delayed capillary refill time. Her chest was clear and abdomen was soft, with no guarding or rigidity. Her Glasgow coma scale was 14 (E3V5M6). Her venous blood gas revealed metabolic acidosis with a lactate of 14.

With a working diagnosis of severe sepsis of unknown source, she was started on broad-spectrum intravenous antibiotics and fluids. As there was no improvement in her haemodynamic status, a referral to the intensive care team was made for inotropic support.

**Fig. 1:**
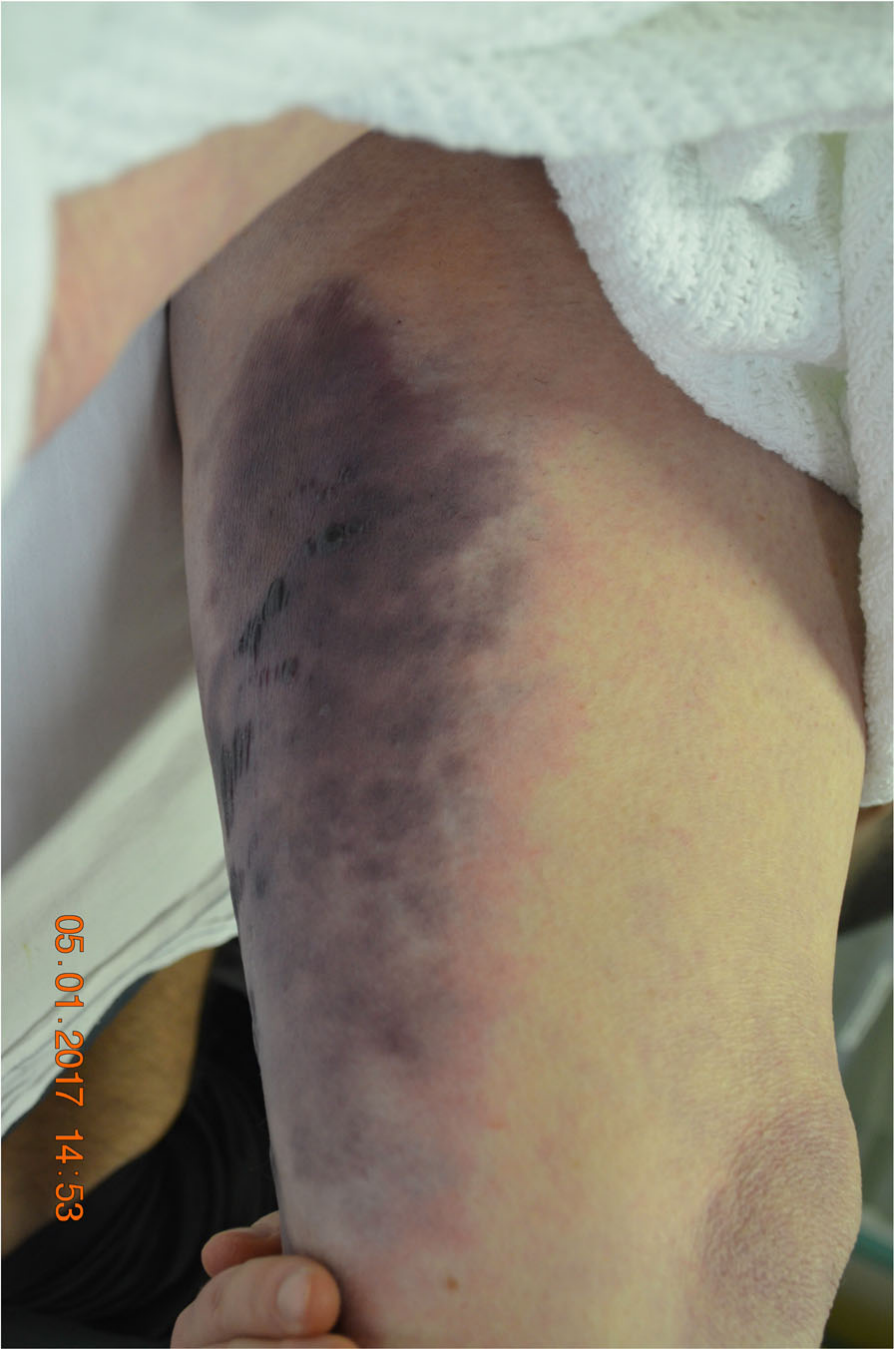
Blackish discolouration with vesicle formation on the thigh

While all of these were being done, she was noticed to have a tender bruise on her leg; however, she denied any trauma or fall. On examination, she had a large area of blackish discolouration and vesicle formation on the posteromedial aspect of the left thigh (Fig. 1), which was tender on palpation. The area looked suspicious of necrotising fasciitis. Her antibiotics were changed as per the microbiology advice to Tazocin and Clindamycin. A urinary catheter was inserted to monitor her fluid balance. An urgent referral to the surgical and orthopaedic team was made for definitive management of surgical debridement. Her initial blood results showed a white cell count of 13.1 and neutrophilia at 11.7. She had a CRP of 439, CPK of 4187 and an AKI stage 3 with urea at 15.2, creatinine of 291 and e-GFR of 13. Serum electrolytes showed a sodium of 137, potassium of 3.4 and chloride of 101.

**Fig. 2:**
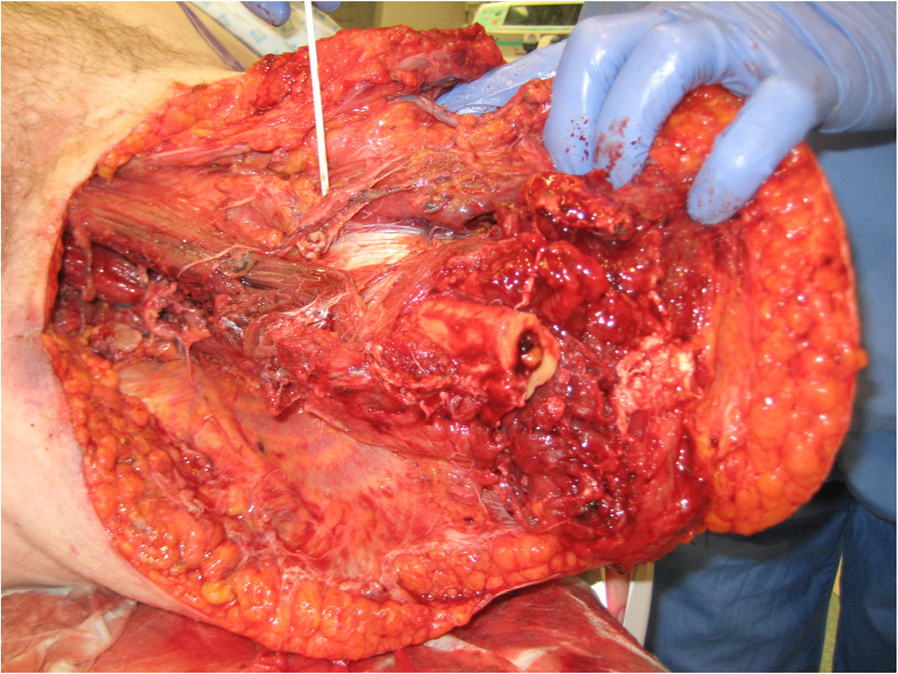
Per-operative image showing necrosis of muscle and soft tissue

**Fig. 3:**
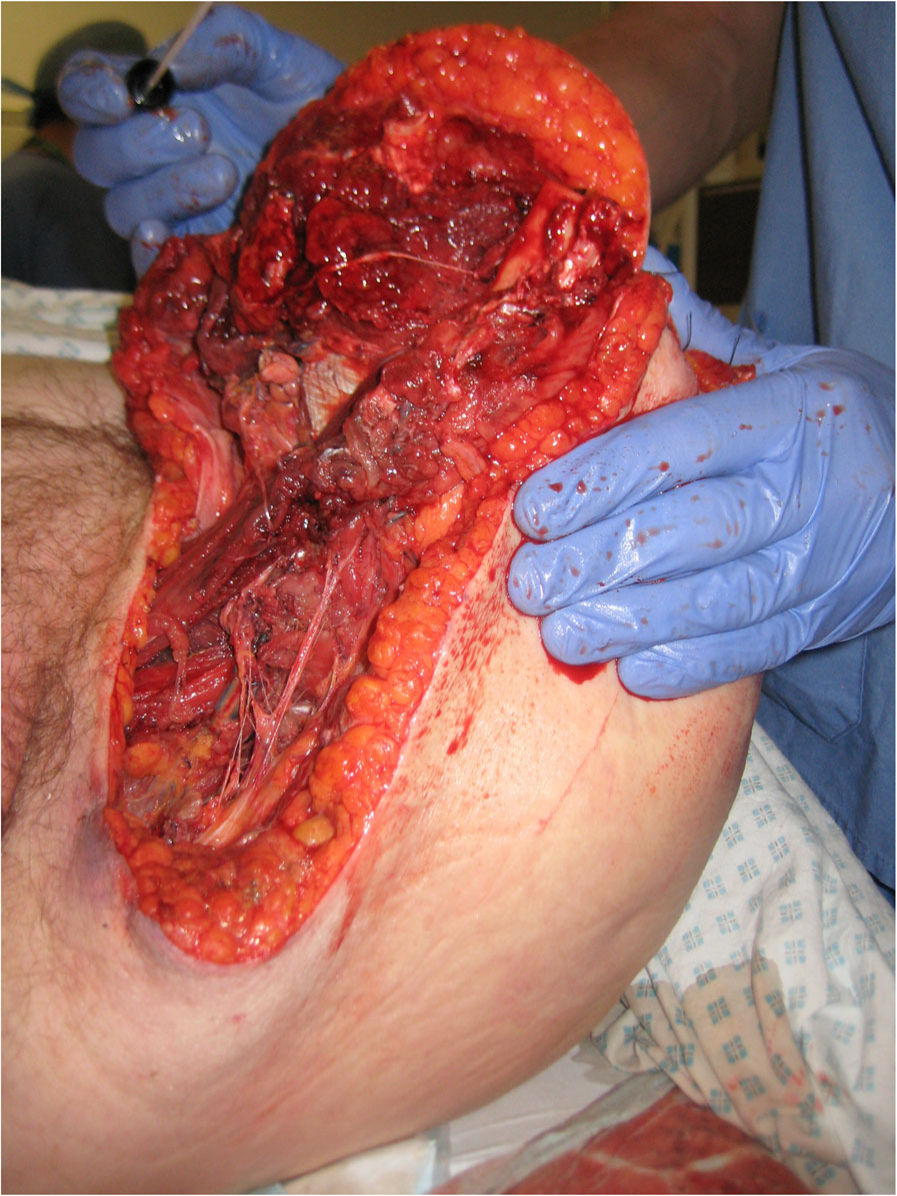
Extent of necrotic tissue up to the pelvis on the second surgery

She underwent debridement of necrosed tissue within few hours of arrival into the emergency department. She subsequently stayed in the intensive care unit and had a further debridement and above-knee amputation of the affected limb (Figs. 2 and 3). On the second surgery, she was found to have necrotic tissue extending up to the pelvis. A subsequent pus culture report confirmed group A Beta haemolytic streptococci as the causative organism. The blood culture showed no growth, and faeces culture was negative and showed no evidence of Salmonella, Shigella, Camplylobacter or *Escherichia coli*. MRSA was not isolated and there was no evidence of C-difficile in the stool.

She eventually died after about 48 h of hospital stay despite an early diagnosis and prompt surgical debridement.

Although necrotising fasciitis is a rare condition, each one of us may still come across a case in our clinical practice. As a learning point from this case, we would like to highlight the importance of a thorough clinical examination of patients with sepsis with no obvious source. As in this patient, the presenting complaint was diarrhoea, which was actually a manifestation rather than a cause for sepsis and clearly had a hidden diagnosis, which could have been missed if the patient was not adequately exposed.

## Discussion

Necrotising fasciitis was first been described in soldiers during the American Civil war in the eighteenth century. This condition has been known by various names including phagadena gangrenosum, Meleney’s gangrene, Fournier’s gangrene (necrotising fasciitis of the perineum). The term necrotising fasciitis was first coined in 1952 by American Surgeon B Wilson.

Necrotising fasciitis is classified into three types based on the organisms involved. Type I is polymicrobial, type II is monomicrobial and type III by a specific pathogen namely marine Vibrio species (*V. vulnificus*). Bacteria associated with type I are a combination of gram-positive cocci (streptococci and staphylococci), gram-negative species (*Escherichia coli*, Acinetobacter, Pseudomonas and Klebsiella) and anaerobic organisms like Bacteroides and Clostridia [1].

NF is a rapidly progressing bacterial infection involving the subcutaneous tissues. The bacteria produce endotoxin and exotoxin [2] that leads to micro-vascular thrombosis [3], tissue ischaemia and liquefactive necrosis. This causes a systemic illness [4] often progressing to septic shock, multi-organ dysfunction and death. The condition is resistant to antimicrobial therapy due to poor drug penetration, hence leaving surgical debridement as the primary treatment option [5].

Previously reported predisposing factors included rheumatoid arthritis, SLE, type 2 diabetes mellitus, renal transplantation, chronic renal failure, injection IV drug use, immunosuppressive therapy and intramuscular injection with nonsteroidal anti-inflammatory drugs. Other factors include trauma, burns, insect bite, chronic skin ulcers and postoperative wound infection. Of these, type 2 diabetes is the commonest underlying medical condition and multiple needle punctures in IV drug users is the commonest trauma predisposing to NF [6].

The clinical picture and presentation of the patient may vary and hence can pose a diagnostic challenge at an early stage and hence requires a very high index of suspicion. The most common early signs are local erythema, warmth, induration, oedema and pain, which are also present in conditions like cellulitis and septic arthritis and hence the diagnostic dilemma. However, pain out of proportion to clinical signs associated with septic shock or failure to respond to broad-spectrum intravenous antibiotics should alert the clinician to possible NF. Patches of skin necrosis, crepitus and bullae with compromised haemodynamic status are alarming signs.

Ultrasound, CT or MRI scan can be done in suspicious cases. Wong et al. [7] devised the Laboratory Risk Indicator for Necrotising Fasciitis (LRINEC) score, which is a valuable diagnostic tool to distinguish necrotising fasciitis from non-necrotising skin infections. LRINEC score ≥ 6 had a positive predictive value of 92% in high-risk patients according to this study. Based on our patient’s initial blood reports, the LRINEC score was 6.

LRINEC score tool

**Table:** 

Variable	Score
C-reactive protein < 150	0
> 150	4
WBC cells/cu mm < 15	0
15–25	1
> 25	2
Haemoglobin g/dl > 13.5	0
11–13.5	1
< 11	2
Sodium mmol/L > 135	0
< 135	2
Creatinine mcg/L < 141	0
> 141	2
Glucose mmol/L < 10	0
> 10	1

Reproduced from: Wong CH, Khin LW, Heng KS; The LRINEC (Laboratory Risk Indicator for Necrotizing Fasciitis) score: a tool for distinguishing necrotizing fasciitis from other soft tissue infections Crit Care Med. 2004, 32: 1535-1541. 10.1097/01.CCM.0000129486.35458.7D.

In suspected or established cases of NF, early surgical exploration and debridement with broad-spectrum intravenous antibiotics is considered key to successful treatment in NF [8]. Surgical exploration can also establish the diagnosis of NF. The operative findings of greyish necrotic deep fascia, lack of resistance to blunt dissection, lack of bleeding of the fascia and presence of foul-smelling ‘dishwater’ pus aids in the confirmation of the diagnosis. Excised tissue should be sent for gram staining, culture and histology. Wound closure is carefully planned as early closure carries the risk of residual infection and poor healing.

## Conclusion

Necrotising fasciitis is a rare life-threatening condition and a surgical emergency. Early diagnosis and prompt treatment with aggressive surgical debridement and intravenous antibiotics are crucial. It requires a multidisciplinary approach at an early stage. High index of suspicion is essential to recognise this life-threatening condition with high morbidity and mortality.

## Abbreviations

AKI: Acute kidney injury
CKD: Chronic kidney disease
CPK: Creatine phosphokinase
CRP: C-reactive protein
LRINEC: Laboratory Risk Indicator for Necrotising Fasciitis
NF: Necrotising fasciitis

## References

[1] Naqvi GA, Malik SA, Jan W (2009). Necrotizing fasciitis of the lower extremity: a case report and current concept of diagnosis and management. Scandinavian Journal of Trauma, Resuscitation and Emergency Medicine.

[2] Hackett SP, Stevens DL (1992). Streptococcal toxic shock syndrome: synthesis of tumor necrosis factor and interleukin-1 by monocytes stimulated with pyrogenic exotoxin a and streptolysin O. J Infect Dis.

[3] Stamenkovic I, Lew PD (1984). Early recognition of potentially fatal necrotizing fasciitis. The use of frozen-section biopsy. N Engl J Med.

[4] Cainzos M, Gonzalez-Rodriguez FJ (2007). Necrotizing soft tissue infections. Curr Opin Crit Care.

[5] Hsiao GH, Chang CH, Hsiao CW (1998). Necrotizing soft tissue infections: surgical or conservative treatment?. Dermatol Surg.

[6] Angoules AG, Kontakis G, Drakoulakis E, Vrentzos G, Granick MS, Giannoudis PV (2007). Necrotising fasciitis of upper and lower limb: a systematic review. Injury.

[7] Wong CH, Khin LW, Heng KS (2004). The LRINEC (laboratory risk Indicator for necrotizing fasciitis) score: a tool for distinguishing necrotizing fasciitis from other soft tissue infections. Crit Care Med.

[8] Edlich RF, Cross CL, Dahlstrom JJ, Long WB. Modern concepts of the diagnosis and treatment of necrotizing fasciitis. J Emerg Med. 2008. https://doi.org/10.1016/j.jemermed.2008.06.024. 10.1016/j.jemermed.2008.06.024 19081698

